# No personalization without participation: on the active contribution of psychiatric patients to the development of a mobile application for mental health

**DOI:** 10.1186/1472-6947-13-78

**Published:** 2013-07-27

**Authors:** Jean-François Pelletier, Michael Rowe, Nathe François, Julie Bordeleau, Sonia Lupien

**Affiliations:** 1 Centre de recherche de l’Institut universitaire en santé mentale de Montréal – affiliated to University of Montreal, 7401, Hochelaga St, Montreal, QC H1N 3M5, Canada; 2 Program for Recovery and Community Health, Department of Psychiatry, Yale School of Medicine, Erector Square, Bldg. One, 319 Peck Street, New Haven, CT 06513, USA

**Keywords:** Personalization, Patient participation, Strategy for patient-oriented research, Information and communication technology, Experiential knowledge translation, Person- centeredness, eMental health, Mobile application, Signature project, Mixed methods

## Abstract

**Background:**

Despite the increasing pervasiveness of mobile computational technologies, knowledge about psychiatric patients’ preferences regarding the design and utility of mobile applications is very poor. This paper reports on a pilot-study that involved 120 psychiatric patients in the development of a mobile application (app) that is being used for data entry into the Signature Project data bank at the *Institut universitaire en santé mentale de Montréal* (IUSMM), Canada. Participants were invited to comment on the ‘look and feel’ of the Signature App. Their input also extended the procedures for data collection. These suggestions may contribute to increased mental health literacy and empowerment of persons with mental illness receiving services at the IUSMM.

**Methods:**

Participants were recruited to fill out a questionnaire on a tablet computer while waiting at the Emergency Room (ER, n = 40), Psychotic Disorders outpatient clinic (n = 40) or Anxiety and Mood Disorders outpatient clinic (n = 40) of IUSMM. Nine patients from each of these sub-groups participated in a focus group to review the results and to discuss how the design and use of the Signature App could be improved to better meet the needs of patients.

**Results:**

This study (n = 120) indicated that psychiatric patients are clearly capable of using a tablet computer to fill out questionnaires for quantitative data entry, and that they enjoyed this experience. Results from the focus groups (n = 27) highlight that the app could also be used by patients to communicate some personal and contextual qualitative information. This would support a holistic and person-centered approach, especially at the ER where people acutely need to describe their recent history and receive emotional support.

**Conclusions:**

This pilot-study has confirmed the necessity of involving patients not only in the testing of a new mobile application, but also as active contributors in the entire research and development process of a person-centered information and communication technology infrastructure. The input of participants was essential in designing the Signature Project computational procedure and making use of the app a positive and empowering experience. Participants also gave critical feedback remarks that went beyond the initial scope of the pilot-study, for example they suggested the addition of a client-clinician component.

## Background

New products are usually designed to match targeted users’ preferences and capacities by involving them in the research and development process (R&D) [1]. In recent years, mobile applications (apps) have become part of daily life for many, and they have also entered the medical field [2]. For example, apps can be used by patients who would like to quit smoking [3] or to lose weight [4], but more often apps are designed to be used by professionals [5]. This may explain why Mosa et al. recently observed that in the scientific literature there is “low coverage” of smart phones apps intended for patients [6] and hence, even less of their eventual contribution to an app R&D process. Of the 14 articles related to mobile applications dedicated to patients’ use, their systematic review captured 2 papers reporting on apps to be used by psychiatric patients, both regarding self-monitoring of substance use [7,8]. Neither of these papers discussed patient contributions to the R&D processes for these apps. Therefore, we still know very little about what the nature of the input from psychiatric patients would be to the R&D of an app to be used by their peers.

In May 2010 the *Centre de recherché de l’Institut univesitaire en santé mentale de Montréal* (CRIUSMM), Canada, aligned itself with the “*four P’s*” of research, according to the National Institute of Mental Health: 1) increasing the capacity to *Predict* who is at risk for developing disease; 2) developing interventions that *Pre-empt* the disease process; 3) using knowledge about individual biological, environmental and social factors for *Personalized* interventions; and 4) ensuring that clinical research involves *Participation* from the diversity of people involved in health care, including patients [9]. This paper mainly focuses on the latter “*P*”, that is on the active participation of IUSMM psychiatric service-users in the R&D of an app imbedded in an innovative computational architecture meant to support more personalized psychiatric interventions (third “*P*”).

A pilot-study was undertaken at IUSMM as a preliminary step in the implementation of a comprehensive data-bank, known as the Signature Project. This endeavour, a first in the field of psychiatric research, aims to embrace use of the “*four P’s*” by collecting a wide array of biological, psychological and social data concerning the 6000 in- and out-patients that receive IUSMM psychiatric care and services and by involving them as active participants. For instance, the psychological and social data sets will be electronically collected by patients filling out a series of questionnaires on a tablet computer with an app (Signature App). To make the Signature App as user-friendly as possible, the pilot-study involved 120 participants who were asked to fill out a questionnaire on a tablet and to comment on this experience. The preliminary results were discussed in focus groups by 27 participants who reflected and gave their insights on how to make this human-computer interaction useful for patients as well as for researchers and clinicians.

In November 2012, the Signature Project officially began to collect psychological and social data from IUSMM patients e.g.: [10-14]. In accordance with the increasing emphasis on the role of patients in developing and evaluating services [15,16], influencing research priorities [17,18], and actively contributing to information and communication technology (ICT) systems design [1,19], this paper reports on the research process and findings, in regard to improving the design and utility of the Signature Project computational procedure and its app suite. The pilot project aimed to answer two questions regarding the participants’ experience using an app: (1) *Are psychiatric patients able to use the mobile application technology?* and (2) *How can the mobile application be improved to better meet patients’ needs?*

### Study design

To assess how psychiatric patients can use an app on a tablet computer to fill out questionnaires and how this data collection procedure can be improved to better meet their needs, 120 psychiatric patients of IUSMM were recruited from October 2010 to January 2011 to complete a questionnaire with a beta version of the Signature App, and to participate in an individual debriefing interview. Participating patients were recruited in specialized outpatient clinics: Anxiety and Mood disorders clinic, and Psychotic disorders clinic, where patients receive services in accordance with these specific diagnostics. Participants at the ER were not recruited on the basis of their diagnostics. Participants were thus selected in three sub-groups: Anxiety and mood disorders (AMD, n = 40), Psychotic disorders (PD, n = 40), and Emergency Room (ER, n = 40). These volumes were chosen in order to generate as many insights as possible and were representative samples of patients treated in the corresponding IUSMM sites. The total volume of 120 patients was in line with comparable studies for broader psychiatric populations [20], and for specific subpopulations such as inpatients suffering from schizophrenia [21-23]. Patients who agreed to participate in the individual testing and debriefing interview were also invited to participate in a focus group. Participation and acceptance ratios are presented in Table 1.

**Table 1:** Participation and acceptance ratios

		**AMD**	**PD**	**ER**	**ALL**
1	Approached for individual testing and interviews	47	55	51	153
2	Recruited for individual testing and interviews	40	40	40	120
3	Accepted to participate in a focus group	19	28	21	68
4	Participated in a focus group	9	9	9	27
5	Ratio of participation for individual testing and interviews	85%	73%	79%	78%
	(Line 2/Line 1)				
6	Ratio of acceptance to participate in a focus group	48%	70%	53%	57%
	(Line 3/Line 2)				

The inclusion criteria were that the participants had to be treated at the testing site, that they had sufficient mental capacities to allow informed consent, and that they were legally able to consent for themselves. At each site, a Research Nurse (RN) was introduced by the clinical team to the patients, unless the chief psychiatrist considered that a patient was not able to participate at that time. The clinical team stopped approaching patients when the target number of participants was reached for each site. Recruitment took approximately two weeks (10 days) at the AMD and PD sites, and four weeks (20 days) at the ER.

Staff in charge at each site identified a liaison clinician, whose responsibility included coordinating the referral of potential participants to the RN. The RN then approached the referred participant and briefly introduced the study. The RN explained that the device would be used to play a video introducing the study and its ethical aspects, followed by an electronic consent form for the participant’s digital signature. Finally, an electronic version of the *Beck Depression Inventory* was administered on the handheld device. The *Beck Depression Inventory* (BDI) is a 21-question self-report inventory [24] that is widely used for measuring the severity of depression. IUSMM Institutional Review Board approval for this pilot-study was granted on September 1^st^, 2010.

## Methods

Six main steps were followed in this pilot-study: (1) identifying and approaching potential participants, (2) introducing the project to them, (3) obtaining their consent, (4) administering the BDI questionnaire with the Signature App, (5) debriefing with each participant, and (6) conducting focus groups. Focus groups were held to further explore the user experience with the Signature App and more broadly to explore ways of enhancing the utility of this computational procedure for patients. The RN was present with the participating patients at all times, and recorded her observations with a template (Table 2: Appendix A of the research protocol). This study was carried out in compliance with the Helsinki Declaration and the Institutional Review Board of IUSMM gave approval (reference number: 2010–012).

**Table 2:** Appendix A of the research protocol

**Themes**	**Observations**
Attention in viewing the video	1) Extremely attentive
	2) Very attentive
	3) Attentive
	4) Not very attentive
	5) Not attentive at all
Feasibility of electronic signature	1) Could sign easily
	2) Had some difficulty to sign
	3) Could not sign/did not want to sign on the app
Understanding the app	1) Had no difficulty in understanding
	2) Did not understand the basic concepts
	3) Could not navigate from question to question
	4) Had some difficulty reading the instructions/questions
Using the app	1) Had no difficulty in using the app
	2) Wanted to press on a button but pressed another one
	3) Tried to press but the software did not detect
	4) Left an answer that did not seem to be wanted answer
Got frustrated	1) No
	2) Yes (if *yes*, why?)
Completed	1) Yes
	2) No (if *no*, why?)

If a patient agreed to view the video, the RN handed the tablet to him or her and started the video. The video lasted approximately 5 minutes, with the Principal Investigator (PI) (on-screen) describing the project’s goal and the ethical aspects of the study that participants must understand before consenting. This step was included to validate the feasibility of explaining the study by video in order to foster informed consent, as patients do not always thoroughly read a multi-page consent form. The presence of the RN throughout the consent process ensured that the participant could, at any time, request further explanation or verbally refuse to participate.

For those who accepted and signed the form, the app automatically transitioned to the BDI questionnaire. For each question, four choices were displayed on the app. The participant simply had to touch the choice that best described them, and the app automatically moved on to the next question.

Once the questionnaire was completed, the RN conducted a short debriefing session with the participant to elicit his/her feedback on their experience of completing the questionnaire, and his/her ideas for improving the intervention. Each session of individual data collection lasted approximately 30 minutes. At the end of the individual session, the participant was invited to attend a focus group to further explore his/her experience with the app. Three focus groups were held, one for each sub-group involved in this study. In each of these subsequent meetings, a facilitator guided 9 participants in a two-hour discussion of their experiences and feelings about their use of the app and their preferences or opinions about how to improve the “look and feel” of the app and the usefulness of the overall computational procedure. To foster discussion, the facilitator asked the participants to comment on the results and themes covered by the individual debriefing sessions (Appendix A of the research protocol used by the RN). Those themes are:

1. Attention in viewing the video;

2. Feasibility of electronic signature;

3. Understanding the app;

4. Using the app;

4.1 Frustration

4.1 Completion

5. Global experience;

6. Maximal duration;

7. Discussing the results.

No names of individual debriefing session participants were mentioned and focus group participants were asked to maintain the confidentiality of other focus group participants.

## Results and discussion

For conversational purposes in focus groups with lay psychiatric patients unfamiliar with specialized statistical terminologies (e.g.: SD), it was necessary to present the results in the most accessible manner to combine quantitative and qualitative data sets. It was thus decided to use percentages in order to foster discussion, as they are presented in Table 3.

**Table 3:** Results by percentages

**Dimensions**	**AMD**	**PD**	**ER**	**ALL**
1- Attention in viewing video	85%	88%	66%	80%
(*…xx% were attentive in viewing the video*)				
2- Feasibility of electronic signature	60%	55%	53%	56%
(*…xx% had no difficulty with electronic signature*)				
3- Understanding the app	88%	90%	93%	90%
(*…xx% had no difficulty with understanding the app*)				
4- Using the app	88%	98%	98%	97%
(*…xx% had no difficulty with using the app*)				
4.1- Frustration	0%	0%	0%	0%
(*…xx% got frustrated or became agitated*)				
4.2- Completion	100%	100%	100%	100%
(*…xx% completed the questionnaire*)				
5- Global experience	86%	90%	87%	87%
(*…xx% had a good experience with the app*)				
6- Maximal duration	62 m	55 m	51 m	56 m
(…*xx minutes could be spent in completing questionnaires on an app*)				
7- Discussing the results	57%	62%	78%	66%
(*…xx% would have liked to discuss the results*)				

### Attention in viewing the video

The level of attention in viewing the video was scored by the RN for each participant on a Lickert scale: 5) extremely attentive, 4) very attentive, 3) attentive with occasional distraction, 2) not very attentive with long periods of distraction, and 1) not attentive/constantly distracted. The scores were summed, for a maximum total of 200 for each group of 40 participants (40X5) and the result was divided by 2 to become a percentage. Rankings for other themes were calculated in a similar fashion.

In focus groups, participants said that they were not surprised by the high level of attention in viewing the video (80%). They suggested that it was normal that the level of attention was lower at the ER due to a person’s typically high level of distress in that situation. Others suggested that the variability in attention might be due to the fact that for some people, it is more convenient to read because reading can be done at one’s own pace, versus hearing a video. Nevertheless, the overall very high attention level indicates that presenting a consent form through video with a multimedia app helps the viewer to stay focused (e.g.: of 40 PD participants, 21 were checked 'extremely attentive' and 13 'very attentive'; 34/40). While waiting at an outpatient clinic, this type of activity is also seen as a positive distraction: it is different and more empowering than simply reading a magazine, for example. Participants suggested that the presence of such a tablet computer could be an incentive to some people to stick to their appointment and that it could be used not only to capture some data but also to enhance mental health literacy by offering accurate and useful information on mental health and mental illness management.

### Feasibility of electronic signature

Electronically signing the consent form was deemed to be feasible, but many holder participants found that it was too different from signing on paper with a pen, and that both signatures did not really resemble. Several participants questioned whether or not such a signature was as legally binding as a penned signature. Also, since participants had to receive a paper copy of their consent form and terms of participation, the RN had to use paper sheets anyway to give the participant a copy and to leave another copy for the hospital record. Thus participants in focus groups recommended use of pen and paper for signing the consent form.

### Understanding and using the app

A small number of participants had some difficulty understanding the Signature App, mainly due to the small size of the font, and received assistance from the RN. Only a few participants had difficulty using the app. Given that 0% became frustrated, as reported by the RN, and that 100% of participants completed the task, it was clear to participants in the focus groups that the Signature App was not only understandable and usable by almost anyone, but that it was in fact fun to use for speedy and easy data entry.

### Global experience

On a scale from 1 to 5, participants were asked to rate their global experience with using the app. They checked 1 when they 'did not like it at all', and 5 when they 'loved it'. Results were again presented to each focus group by percentage with regards to the maximum possible total for that group. There was very little difference between the groups’ responses, with patients from the PD group (90%) enjoying it 4% more than the AMD group (86%) and 3% more than the ER group (87%). Study participants were generally very satisfied, but one common concern was about the BDI itself. Many would have liked to be able to add their own answer when it was not among the offered choices. The BDI does not allow that. Also, having a function such as a text box that would appear after completion of each [or after all] responses would allow people to note their impressions or comments, and might enhance participant’s interactive experience with the app.

### Maximal duration

We wanted to know how much time service-users thought their peers could dedicate to using an app to fill out electronic forms. Some said “no more than 10 minutes,” others said “endlessly.” Some participants gave a range, for example “45 minutes to an hour”. In such cases, the higher figure in the range was used for data entry. The longest time period given was 4 hours (240 minutes). When the answer was “endlessly,” 240 minutes was used for data entry.

The average for all 120 participants was 56 minutes (Table 3). To focus group participants, it thus seemed reasonable to expect future respondents to be able and willing to dedicate about an hour of their time. Participants also suggested, however, that there could be a great deal of variability among users. People in acute distress might not be able to remain focused for more than 15 minutes. Therefore, one recommendation was to allow respondents to choose, among different sets of questions, which ones they would like to fill out first. Participants felt that future respondents should be made comfortable to complete only one set, if that was all they could do at that time, and then as many as they could in their preferred order.

### Discussing the results

This pilot-study evaluated only the Signature App performance in administering electronic consent and questionnaires and the overall experience with the tablet, not the Beck Depression Inventory in particular. Nevertheless, participants were asked if they would have liked to discuss the results of the Beck Depression Inventory if recorded. There was some variability among groups in a range from 57% of AMD participants to 78% of ER participants saying that they “would have liked” or “maybe would have liked” to discuss the results. Some participants said that it would depend on whom they would be discussing the results with, for example in identifying topics of personal interest to be discussed with the clinician.

ER participants were more inclined to discuss the results right away with their clinician, while being the least attentive to viewing the video. There was a difference of 22% between the ER and PD groups with *attention in viewing the video* and a difference of 21% between the ER and the AMD groups with *discussing the results*. The ER focus group participants proposed the hypothesis that the more a patient is in need of speaking to someone; the less they would pay attention to a video. However, this could also be true with information and consent paper forms. ER patients would thus be more in need of interaction when in quest for emotional support.

When asked what the implication would be for the app designers and developers, the ER focus group formulated the recommendation of adding a function to the multimedia app that would allow people to tell a bit of their story before meeting their clinician. Participants were aware that even so, it was possible that the clinician could not have time to immediately discuss the specific issue(s) some patients would like to raise but in the least, these patients could tell their story, and the clinician could listen to these stories afterward. Some participants said it might be very frustrating not to have this opportunity at all in the ER. They recommended the addition of a question about recent life events, a question that could be answered verbally and audio-recorded: *What brought you here?* This would also be a good way to optimize the multimedia bi-directional capabilities of a mobile application.

### Implementation of recommendations

In November 2012, the Signature Project was officially launched at IUSMM, with Institutional Review Board approval. The Signature computational procedure and app interface were specifically designed for maximum usefulness among populations suffering from cognitive deficits and psychiatric disorders, a group that was included in the R&D process of the Signature App. Literature underlines the importance of actively involving users in the system design process both to influence design and for the significant cost-benefit advantages of such an approach, compared to when targeted users test a new product only when it is ready to be commercialized and that there is too little room for improvements [25,26]. This pilot study and contextual inquiry [27] aimed at gaining a better understanding of participants’ preferences with regards to the human-machine interaction and use of the app. Almost all patients’ recommendations that emanated from the focus groups were addressed, as discussed below.

For this pilot-study, the PI and first author of this paper appeared in the video to explain the electronic data collection process, research objectives, and the consent process. Participants liked to hear and look at someone explaining the project, but in focus group it was recommended to provide information and consent paper form to whoever would prefer to read (attention in viewing the video). As recommended in focus groups, it was also decided to use typical pen-paper consent form (feasibility of electronic signature). Again as recommended, it is now possible to change the size of the font displayed on the screen to facilitate the reading of the questions (understanding and using the app), but it is not yet possible to leave comments for enhanced interactivity (global experience). Nor is it possible, at present, for participants to choose the questionnaires they would prefer to complete first. They can, however, stop at any time and their choice of answers is kept for aggregation, even if each of the actual 168 questions are not all answered. Based on participant feedback, it is anticipated the average time for completion of the questions is one hour (maximal duration). Finally, at the time of inauguration of the Signature Project, it was not planned that patients would necessarily discuss their results of the questionnaires with their clinician, but they will know, per the information and consent form, that their clinician does have access to these results. It will therefore be possible for patients to ask their clinician to discuss the results of their participation with them. As highlighted by Bauer and Moessner, the increasing availability of Information and Communication Technologies has opened new perspectives for prevention, self-help and treatment of mental disorders (“e-mental health”), as well as within the context of regular face-to-face care [28]. This study tends to support the view that the app can be used to foster greater interactivity within a regular face-to-face clinical encounter.

More than 25 years ago, Norman and Draper [29] emphasized the importance of having a good understanding of the users’ expectations regarding a new technological product, but without necessarily involving them actively in the R&D process. Since then, active user participation throughout the entire development process and throughout the system lifecycle has become a constitutive characteristic of user-centered systems design [30]. Redesigning an app to match the expectations and values of psychiatric patients involved more than feedback on the app’s colors and buttons: encompassed comments on the quality of the service and how this app could be integrated into service delivery and organizational processes [31,32]. Usually, new mobile applications are designed to be used by professionals [5], and with a predominately individual-based approach [33]. However, “individualized” does not equal “personalized.” When an app is designed to be used by patients and when patients are invited to give their input about the app’s design and potential use, questioning how the app will impact the person’s interaction with others (particularly clinicians) is necessary. An implication for developers of mobile applications who seek input from psychiatric patients is that they should be prepared to respond to potentially sensitive questions, as participants may want to discuss matters that go well beyond the mechanics needed to provide data. For example, some patients with schizophrenia believe that use of the Internet has the potential to favorably change their relationships with doctors [19], and this could be the case for mobile applications as well.

#### Study strengths and limitations

The participation of psychiatric patients as active contributors is part of the very foundation and history of the Signature Project, having proven its utility in the R&D process. Their input was sought and taken into account. Still, a fully person-centered approach would have included other stakeholders in the development of the app, since clinical and interpersonal dynamics can be affected by the introduction of the app in clinical practice. For this reason, Progmet, Georgiou and Westbrook suggested that implementers and adopters of such technologies should explicitly address questions about why and how the mobility of these devices is expected to improve care delivery and to support the work of clinicians [34]. Differences in interaction seem to predict adherence to what would be agreed upon with a clinician [35]. Therefore, an important limitation of this pilot-study is that clinicians whose practice might be impacted were not as involved in this pilot-study, from the onset, as were patients. This could be corrected with a follow-up study similar to this one with some IUSMM clinicians, as the Signature App will continue to evolve from one iteration to the next.

Another limitation is that only one questionnaire was used in this study, the BDI, while several more will be administered with the full Signature Project. It is possible that patients will not be as interested in discussing results with their clinician after having spent an hour or more with an app, compared to having spent only a few minutes doing so, as was the case for this study. Also, as its name indicates, the BDI is solely focused on depression, and this may limit the generalizability of findings. It is possible that the results would differ for other types or levels of psychological distress.

## Conclusions

The use of electronic devices to answer psychiatric questionnaires is certainly not new, for example in the assessment of anxiety [36,37]. What is new with this study is that it provides insight into the added value of involving psychiatric patients early on as active contributors in the development of a mobile application. This study revealed that psychiatric patients can clearly manipulate a tablet computer for data entry while waiting at a clinic or even at the ER, and that this technology is appealing to most of them. The active participation and input of patients to this pilot-study were essential in making the Signature Project data collection procedure and app a positive, empowering and genuinely personalized experience. Participants’ recommendations were taken into consideration to improve their experience of human-computer interaction. Results of this pilot-study can support informed decisions about how to involve psychiatric patients in the R&D of an app that would facilitate data entry. More importantly, it suggests that an R&D process should anticipate the clinical interaction in the assessment of potential outcomes over the quality of mental health care. In that respect, more participatory research is needed by involving clinicians as well as patients in the R&D of an app to be used by patients and providers through the clinical encounter. Family members and other significant others of patients could be involved too.

Many apps that are currently available do not have written privacy policies [38]. This should soon cease to be the case, as emerging certification standards now request such policies, particularly in the medical field. Documentation about how the content of an app was formulated should also be provided to make sure that this content is reliable [39]. As there can be several categories on a continuum of possible levels and degrees of participation [40], among those standards, we suggest that information should also be provided about how and to what extent the targeted users of an app, whether clinicians or patients, were included in the R&D process of that app and interacted.

## Competing interests

The authors declare that they have no competing interests.

## Authors’ contributions

JFP carried out the pilot-study, performed the qualitative analysis and drafted the manuscript. JB and MR helped to draft the manuscript. NF supervised the implementation of the Signature Project and SL is the Principal Investigator of the Signature Project and Scientific Director of the CRIUSMM. All authors read and approved the final manuscript.

## Pre-publication history

The pre-publication history for this paper can be accessed here:

http://www.biomedcentral.com/1472-6947/13/78/prepub
